# Identification of novel snoRNA-based biomarkers for clear cell renal cell carcinoma from urine-derived extracellular vesicles

**DOI:** 10.1186/s13062-024-00467-0

**Published:** 2024-05-13

**Authors:** Konrad Grützmann, Karsten Salomo, Alexander Krüger, Andrea Lohse-Fischer, Kati Erdmann, Michael Seifert, Gustavo Baretton, Daniela Aust, Doreen William, Evelin Schröck, Christian Thomas, Susanne Füssel

**Affiliations:** 1 Core Unit for Molecular Tumor Diagnostics (CMTD), National Center for Tumor Diseases Dresden (NCT/UCC), 01307 Dresden, Germany; 2 German Cancer Research Center (DKFZ), 69120 Heidelberg, Germany; 3 German Cancer Consortium (DKTK), 69120 Heidelberg, Germany; 4 Institute for Medical Informatics and Biometry, Faculty of Medicine Carl Gustav Carus, Technische Universität Dresden, 01307 Dresden, Germany; 5 Department of Urology, Faculty of Medicine Carl Gustav Carus, University Hospital Carl Gustav Carus, Technische Universität Dresden, 01307 Dresden, Germany; 6 Institute for Pathology, Faculty of Medicine Carl Gustav Carus, University Hospital Carl Gustav Carus, Technische Universität Dresden, 01307 Dresden, Germany; 7 Institute for Clinical Genetics, Faculty of Medicine Carl Gustav Carus, University Hospital Carl Gustav Carus, Technische Universität Dresden, 01307 Dresden, Germany; 8 Institute of Molecular Cell Biology and Genetics, ERN GENTURIS, Hereditary Cancer Syndrome Center Dresden, Max Planck, 01307 Dresden, Germany

**Keywords:** Biomarker, Cancer diagnostics, Clear cell renal cell carcinoma, Exosomes, Extracellular vesicles, Kidney cancer, Liquid biopsy, snoRNA, Transcriptional biomarker, Urine

## Abstract

**Background:**

Clear cell renal cell carcinoma (ccRCC) is the most common subtype of RCC with high rates of metastasis. Targeted therapies such as tyrosine kinase and checkpoint inhibitors have improved treatment success, but therapy-related side effects and tumor recurrence remain a challenge. As a result, ccRCC still have a high mortality rate. Early detection before metastasis has great potential to improve outcomes, but no suitable biomarker specific for ccRCC is available so far. Therefore, molecular biomarkers derived from body fluids have been investigated over the past decade. Among them, RNAs from urine-derived extracellular vesicles (EVs) are very promising.

**Methods:**

RNA was extracted from urine-derived EVs from a cohort of 78 subjects (54 ccRCC patients, 24 urolithiasis controls). RNA-seq was performed on the discovery cohort, a subset of the whole cohort (47 ccRCC, 16 urolithiasis). Reads were then mapped to the genome, and expression was quantified based on 100 nt long contiguous genomic regions. Cluster analysis and differential region expression analysis were performed with adjustment for age and gender. The candidate biomarkers were validated by qPCR in the entire cohort. Receiver operating characteristic, area under the curve and odds ratios were used to evaluate the diagnostic potential of the models.

**Results:**

An initial cluster analysis of RNA-seq expression data showed separation by the subjects’ gender, but not by tumor status. Therefore, the following analyses were done, adjusting for gender and age. The regions differentially expressed between ccRCC and urolithiasis patients mainly overlapped with small nucleolar RNAs (snoRNAs). The differential expression of four snoRNAs (*SNORD99*, *SNORD22*, *SNORD26*, *SNORA50C*) was validated by quantitative PCR. Confounder-adjusted regression models were then used to classify the validation cohort into ccRCC and tumor-free subjects. Corresponding accuracies ranged from 0.654 to 0.744. Models combining multiple genes and the risk factors obesity and hypertension showed improved diagnostic performance with an accuracy of up to 0.811 for *SNORD99* and *SNORA50C* (*p* = 0.0091).

**Conclusions:**

Our study uncovered four previously unrecognized snoRNA biomarkers from urine-derived EVs, advancing the search for a robust, easy-to-use ccRCC screening method.

**Supplementary Information:**

The online version contains supplementary material available at 10.1186/s13062-024-00467-0.

## Background

There are more than 330,000 new cases of renal cell carcinoma (RCC) and 140,000 related deaths worldwide each year [1]. Clear cell renal cell carcinoma (ccRCC) is the most common subtype of RCC and is typically asymptomatic in its early stages [2]. However, 30% of newly detected cases are already metastatic [3], resulting in low survival rates [4]. CcRCC is resistant to radio- and chemotherapy [1] and often recurs after nephrectomy [5]. Targeted therapies have been developed over the past decades [6]. Older, low-response immunotherapies for metastatic RCC [7] have been replaced by tyrosine kinase inhibitors and mTOR inhibitors [8–11]. New generation checkpoint inhibitors show improved efficacy in RCC treatment [12–14]. However, not all patients respond well and side effects are possible [15]. Despite prolonged survival, most patients experience tumor progression over time [16]. Sub-classification with treatment response prediction is essential to advance patient care [15]. A prognostic panel for ccRCC [17] and a panel for potential adjuvant therapy decision in RCC [18] were developed. However, pre-metastatic diagnosis of ccRCC bears the greatest potential to improve patient outcomes and to reduce the financial and emotional burden of the disease.

Computed tomography (CT) is inappropriate for the diagnosis of RCC due to frequent false-positive and incidental findings [19]. Ultrasound is a less expensive and well tolerated option, but has an overall lower accuracy and reduced ability to detect small RCCs [19]. More specific molecular biomarkers have been identified, but they are often less accurate than CT and are laborious and expensive. Other drawbacks include reduced sensitivity with regard to tumor size [20, 21] and poor discrimination of benign tumors [22]. Thus, there is a need for cheaper, non-invasive screening methods that are more accurate and easier to use for early detection of ccRCC and effective therapy initiation [19].

Extracellular vesicles (EVs) are circulating particles in bodily fluids that carry RNA, DNA, proteins and lipids from their host cell [23]. Exosomes, a subtype of EVs, develop in the endosomal system [23]. EVs are taken up by recipient cells and play a significant role in cellular information exchange, particularly in the tumor microenvironment, affecting fibroblasts, endothelial, immune and cancer stem cells [24, 25]. EVs elicit functional responses and mediate cellular properties [23]. They are involved in tumorigenesis, metastasis and immune evasion [26], with tumorigenic EVs inducing signaling and phenotypic changes in the recipient cells through RNA shuttle [27–29].

EVs are a promising source for biomarker discovery due to their stability, accessibility and specific content [30]. Several miRNAs from serum- and plasma-derived EVs have shown diagnostic potential for ccRCC, including miR-210, miR-1233 and miR-224 [31–33]. Kidney epithelium-derived EVs enter the urinary tract and are found in patient urine. In principle, they may reflect the molecular pathologic state [30]. However, corresponding research has only started [34]. Urinary EV-derived miRNAs, such as miR-30c-5p and miR-205, have shown potential as biomarkers [35, 36] and combinations of urinary EV-derived miRNAs can differentiate healthy subjects from those with benign renal tumors and early-stage or advanced ccRCC [37]. Besides miRNAs, small nucleolar RNAs (snoRNAs) hold promise as biomarkers for several types of tumors including ccRCC [38]. Additionally, studies have explored lipids and proteins from urinary EVs as potential biomarkers for RCC [39, 40]. Urine EV-derived biomarker assays have the potential to support screenings for ccRCC due to the non-invasive and pain-free nature of the “liquid biopsy”.

To verify this hypothesis, we investigated urine-derived EVs in a cohort of 78 subjects (54 ccRCC and 24 urolithiasis patients). Regarding clinical diagnoses, urolithiasis is a more relevant control condition than healthy subjects. We sequenced and screened RNA transcripts outside the usual miRNA realm. We evaluated the differentially expressed candidate RNAs for their potential to classify ccRCC and tumor-free subjects. Our study uncovered a small set of so-far unexplored snoRNAs as gender- and age-controlled biomarkers. This is an advance in the search for a robust urine EV-derived RCC screening method.

## Materials and methods

### Cohort

**Fig. 1:**
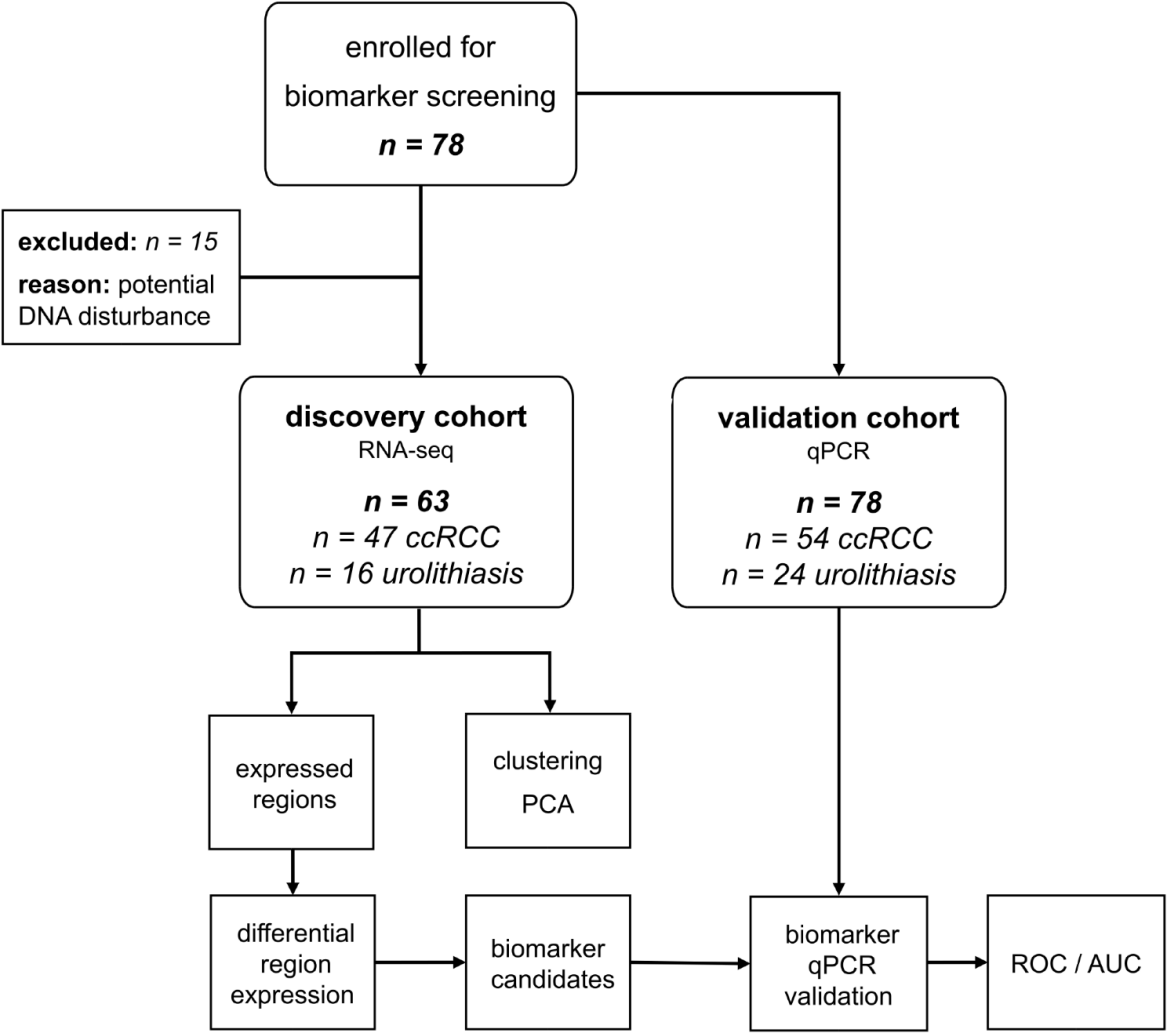
Flow chart of cohort selection and study design. AUC – area under the curve, ccRCC - clear cell renal cell carcinoma patients, PCA – principal component analysis, PCR – polymerase chain reaction, ROC – receiver operating characteristics, urolithiasis – control patients

Preoperative urine samples were collected from patients with ccRCC undergoing partial or total nephrectomy at the Department of Urology at the University Hospital Dresden, Germany, between May 2014 and July 2017. Patients with urolithiasis, who donated spontaneous urine before any intervention, served as tumor-free control group. A total of 78 subjects (54 ccRCC patients and 24 controls) were analyzed in the study. Samples with remnants of DNA (defined as a DNA/RNA ratio > 0 and a coverage variance < 0.5) were removed from the discovery cohort (7 ccRCC patients and 8 controls). Thus, the discovery cohort (*n* = 63) consisted of samples from 47 ccRCC patients and 16 controls (Fig. 1) that underwent RNA-Seq. Nevertheless, all 78 initial subjects were included in the validation cohort for expression analysis by quantitative PCR (qPCR). The distribution of gender, age, presence of obesity and hypertension as well as TNM stage of the ccRCC patients and urolithiasis controls as well as information on urine samples and RNA yield are shown in Tables 1 and 2 and Suppl. Table S1.

**Table 1:** Demographic, clinicopathological and technical characteristics of the included ccRCC patients

		Discovery cohort (RNA-seq)		Validation cohort (qPCR)	
Parameter	Category	Number [n]	Percentage [%]	Number [n]	Percentage [%]
gender	male	36	77	36	67
	female	11	23	18	33
age (years)	median (range)	64 (40–80)		65 (40–80)	
obesity (BMI ≥ 30)	yes	12	26	16	30
	no	35	74	38	70
hypertension	yes	30	64	33	61
	no	14	30	17	31
tumor stage	pT1	32	68	38	70
	pT2	4	9	5	9
	pT3	9	19	9	17
	pT4	2	4	2	4
Lymph node stage	c/pN0	23	49	28	52
	c/pN1	2	4	2	4
	c/pNx	22	47	24	44
Metastasis stage	c/pM0	21	45	27	50
	c/pM1	2	4	2	4
	c/pMx	24	51	25	46
tumor grade	G1	7	15	10	19
	G2	32	68	33	61
	G3	6	13	9	17
	G4	2	4	2	4
urine volume (ml)	median (range)	60 (20–110)		59 (20–110)	
RNA yield (ng)	median (range)	29 (7-304)		32 (7-2196)	

The RNA-seq discovery cohort comprised *n* = 47 of 63 and the qPCR validation cohort *n* = 54 ccRCC patients of a total of 78 test subjects. The table shows the absolute and relative distribution of gender, age and clinicopathological parameters

**Table 2:** Demographic and technical characteristics of the tumor-free control subjects with urolithiasis

		Discovery cohort (RNA-seq)		Validation cohort (qPCR)	
Parameter	Category	Number [n]	Percentage [%]	Number [n]	Percentage [%]
gender	male	15	94	20	83
	female	1	6	4	17
age (years)	median (range)	63 (43–77)		62 (43–77)	
obesity (BMI ≥ 30)	yes	3	19	4	17
	no	13	81	20	83
hypertension	yes	10	63	14	58
	no	6	38	10	42
urine volume (ml)	median (range)	58.5 (30–100)		58.5 (30–100)	
RNA yield (ng)	median (range)	27 (14-1309)		44 (14-3234)	

The control group included *n* = 16 patients with urolithiasis of 64 test subjects in the RNA-seq discovery cohort and *n* = 24 patients with urolithiasis of 78 test subjects in the qPCR validation cohort

### Enrichment, validation and RNA-Seq of urinary EVs

Collected urine specimens (*n* = 78) with a mean volume of 59 ml (range 20–110 ml) were kept on ice and processed within 2 h after collection as previously described with minor modifications [41]. After an initial centrifugation for 10 min at 1500 g and 4 °C, the urine supernatants were frozen at -80 °C until further processing. After thawing, 8 ml of urine supernatant were centrifuged for 5 min at 3200 g and 4 °C. A total of 7 ml of this centrifuged supernatant was incubated overnight at 4 °C, with 2.1 ml precipitation buffer from the miRCURY Exosome Cell/Urine/CSF Kit (Qiagen). After two subsequent centrifugations (1st for 30 min and 2nd for 5 min) at 3200 g and 4 °C the pellet containing enriched urinary EVs was lysed in 1 ml Qiazol (Qiagen) and stored at -80 °C until isolation of exosomal RNA. This was accomplished using the Direct-zol RNA MiniPrep Kit (Zymo Research) according to Fuessel et al. [41]. Finally, the RNA was eluted with 50 µl nuclease-free water and subjected to a quantity and quality control. The yield, purity and integrity of the exosomal RNA were analyzed with a Fragment Analyzer (Agilent Technologies) and the High Sensitivity RNA Analysis Kit (DNF-472, Agilent Technologies). The median RNA yield was 19 ng (range 4–1797 ng). At least 1 ng RNA were used for expression analyses by RNA-seq.

Parallel preparations of urinary EVs were used for assessment of exosomal proteins (Alix, CD9, CD63, CD81, FLOT1, TSG101) by Western blot. Calnexin (CANX) served as a control marker for the endoplasmic reticulum, which should be absent in exosome preparations (Suppl. Table S2). Additionally, nanotracking analysis was performed on the Zeta View instrument according to the manufacturer’s recommendations (Particle Metrix GmbH) to assess the concentration and size distribution of the enriched EVs.

Sequencing libraries were prepared using the SMARTer smRNA Seq Kit (TaKaRa Bio Europe SAS) without final size selection. The barcoded libraries were pooled and sequenced 50 bp single-end on a NextSeq500 (Illumina) with High Output 75 bp flow cells.

### Read trimming and decontamination

Reads were converted from bcl to fastq format using bcl2fastq (v2.17.1.14), allowing for one barcode mismatch, and then trimmed for adapters and quality using cutadapt (v2.4) [42] based on recommendations from TaKaRa: cutadapt -nextseq-trim = 15 -m 15 -u 3 -a AAAAAAAAAA. Reads were then cleaned from potential contamination by other species using FastQ Screen v0.14.0 [43]: -conf config.file -nohits -aligner bowtie2–force, where config.file specifies the genomes against which screening was performed. Next, bowtie2 genome indices provided by FastQ Screen were used: sequencing adapters, PhiX, *E. coli*, *S. cerevisiae*, lambda phage, diverse mitochondria, diverse rRNA, diverse vectors. Additional species genomes were searched and downloaded on 2019/09/23 as follows: 1529 representative bacteria from NCBI Assembly (query: Search all[filter] AND bacteria[filter] AND “latest refseq“[filter] AND “complete genome“[filter] AND “representative genome“[filter] AND (all[filter] NOT “derived from surveillance project“[filter] AND all[filter] NOT anomalous[filter] ); 328 archaea from NCBI Assembly (query: Search all[filter] AND archaea[filter] AND “latest refseq“[filter] AND “complete genome“[filter] AND ( all[filter] NOT “derived from surveillance project“[filter] AND all[filter] NOT anomalous[filter] )); 280 fungal species from NCBI RefSeq, and from Ensembl: barley (Hordeum_vulgare.IBSC_v2), wheat (Triticum_aestivum.IWGSC), and from NCBI: maize (Zea mays), rice (Oryza sativa), sorghum, soybean (glycine_max), grape (vitis_vinifera). The genomes were indexed using bowtie2 with default parameters.

### Alignment and expression quantification

Clean reads were then aligned to the 1000 Genomes Project Phase II reference, including the d5 decoy sequences (hs37d5) and the Gencode annotation (GRCh37.p13), using STAR (v2.5.2b) [44] in 2-pass mapping mode. Splice junctions from the first mapping pass were inserted as a guide for the second pass. Stricter than default mapping parameters were used: --alignIntronMax 1 --outFilterMismatchNmax 1 --outFilterMatchNmin 16 --outFilterMatchNminOverLread 0 --outFilterScoreMinOverLread 0 --outFilterMismatchNoverLmax 0.03. Uniquely mapped reads with a minimum of 25 match positions and a maximum of 10% deletions, insertions or soft-clipped positions were retained. Samples with DNA signals were excluded from the discovery cohort when DNA concentration was > 0 and coverage-variance (CV) was < 0.5. We calculated sample-wise CV as follows: divide all chromosomes into tiles of 1 M bases, quantify the standard deviation and mean read coverage of the tiles (samtools bedcov) for each chromosome, then divide the median of the standard deviations by the median of the mean coverages. In the case of DNA sequencing, coverage is expected to be very uniform across chromosomes, while coverage in RNA sequencing (RNA-seq) varies widely as it reflects gene expression. We considered CV as an indicator of sufficient RNA concentration in the sample (CV vs. DNA/RNA concentration ratio: Pearson *R* = -0.38, *p* = 0.0004). The CV of the entire cohort ranged from 0.2 to 1.6. This resulted in a discovery cohort of 47 ccRCC and 16 urolithiasis control patients. We found that reads rarely covered entire exons or genes and therefore analyzed transcribed regions instead. For this purpose, we divided the whole genome into 30,956,785 contiguous regions of 100 nt and counted the number of reads mapped to each region for each sample using bedtools [45]. We later analyzed only regions with evidence of transcription defined as having at least five mapped reads in at least 20% of the discovery cohort.

### Cluster analysis and differential region expression

Principal component analysis was performed with the regularized log-transformation of transcripts per kilobase per million (rlog TPM) values of all expressed regions using the R package stats. Clustering was performed using Euclidean distance and complete linkage with the rlog TPM values. Heatmaps were plotted using the R package ComplexHeatmap [46] and hclust from the R stats package (v3.4.2) [47]. DESeq2 (v1.10.1) [48] was used to identify differentially expressed regions between ccRCC and urolithiasis patients, adjusting for gender and age of the subjects. Only regions with p-values adjusted for multiple testing < 0.05 were retained. Regions were annotated with names and descriptions of the overlapping genes from Ensembl version GRCh37, v75.

### Screen for suitable qPCR reference genes and qPCR validation

The RNA-Seq TPM values of all expressed regions were screened for suitable PCR reference genes that were stably expressed in all samples. For this purpose, TPM means and standard deviations (SD) of the regions were calculated and screened for consecutive regions with a high mean but a low SD/mean ratio for which TaqMan gene expression assays (Thermo Fisher Scientific) were available or could be designed. This yielded reference genes *ACTB* and *RNY3* (Suppl. Table S3).

The different snoRNA candidates and the respective reference genes were quantified on a LightCycler 480 Real-Time PCR System (Roche Diagnostics). On average 100 ng of RNA were reverse transcribed using MultiScribe Reverse Transcriptase or SuperScript III Reverse Transcriptase (Thermo Fisher Scientific), depending on the intended qPCR assay type (Suppl. Table S3). The resulting cDNA product was preamplified using a mixture of the specific TaqMan gene expression assays and the TaqMan PreAmp Master Mix according to manufacturer’s recommendations (Thermo Fisher Scientific). Each qPCR reaction (final volume 10 µl) consisted of 1 µl of the 2- or 3-fold diluted cDNA preamplificate, the respective TaqMan gene expression assay, GoTaq Probe qPCR Master Mix (Promega), and nuclease-free water. The qPCR reaction was set up as follows: 10 min initial denaturation at 95 °C, 45 cycles of 15 s denaturation at 95 °C and 1 min annealing / extension at 60 °C. The threshold cycles (CT) determined by the second derivative method were averaged from two independent reactions for each transcript per sample. Subsequently, the delta-delta-CT method was used to calculate the relative snoRNA levels normalized to the respective reference RNAs. Due to the different design of the TaqMan gene expression assays for the different snoRNAs, only reference gene assays of the same design could be used (Suppl. Table S3). Thus, the expression of the snoRNAs *SNORD22*, *SNORD26* and *SNORA81* was normalized to that of the reference gene *RNY3*. For the snoRNAs *SNORA50C* and *SNORD99* the reference gene *ACTB* was used for normalization.

### Receiver operating characteristic analysis

Relative expression levels for candidate transcripts obtained by qPCR were examined for their predictive power using a generalized linear model (glm function of stats/R). For this purpose, the molecule counts were divided by the counts of the respective reference gene, logarithmized and then used as predictors with the covariates gender and age as well as hypertension and obesity (BMI ≥ 30), where applicable. Receiver operating characteristic (ROC) curves and the area under the curve (AUC) were calculated using the R package pROC (1.17.0.1) [49] and plotted using ggplot2 (3.3.0) [50]. Odds ratios for the risk of ccRCC were calculated on the basis of a transcript expression change in the size of the interquartile range of the respective gene. We assessed the model fit by comparing its deviance with that of a null model containing only the intercept, using a one-tailed chi-squared test.

### Cross-validation to asses model performance

A fivefold cross-validation was performed to assess the robustness of the regression model results. Therefore, the validation cohort was randomly divided into five bins of at least 10 ccRCC and four urolithiasis patients each, reflecting the proportions of the full cohort. Each model was trained on four data bins and tested on the remaining data bin. Training and testing were repeated on all possible combinations of the split data. The entire procedure was repeated 1000 times for each model to calculate means and standard deviations of the performance metrics.

## Results

### RNA from a small fraction of the genome is found in urine-derived EVs

A total of 78 subjects (54 ccRCC patients and 24 controls) were analyzed in the study, where urolithiasis cases are a clinically more relevant control condition than healthy subjects. EVs were extracted from patients’ urine and characterized by size and quality measurements. Western blot analysis revealed that they displayed typical exosomal markers such as Alix, CD9, CD63, CD81, FLOT1 and TSG101. Calnexin, a marker of the endoplasmic reticulum, was absent (Suppl. Figure S1). Moreover, the EV preparations exhibited a size distribution and diameters with a peak around 110–120 nm typical for exosomes (Suppl. Figure S2). Nevertheless, exosomes and other microvesicles are often extracted at the same time and difficult to separate specifically [26]. Therefore, we use the term EV here for simplicity.

The urinary EV samples were subjected to small RNA transcriptome sequencing. Samples with potential DNA disturbance were removed from the discovery cohort (*n* = 63), which then consisted of 47 ccRCC patients and 16 controls (Fig. 1; Tables 1 and 2, Suppl. Table S1). Quality trimmed and filtered RNA-Seq reads were aligned to the human genome. To exploit a wider range of exosomal RNA, we did not restrict the analyses to known transcripts but screened for expressed regions of the human genome. We found that 6234 (0.02%) of the nearly 31 million 100 nt long genomic regions were expressed with at least five detected reads in at least 20% of the cohort. The expression values of the genomic regions were further subjected to clustering and differential expression analysis. Although RNA exclusion from cells via EVs more accurately reflects the underlying biological process we simply write genomic region expression in the following.

### Cluster analysis showed that RNA profiles strongly reflect gender of the urine donors

**Fig. 2:**
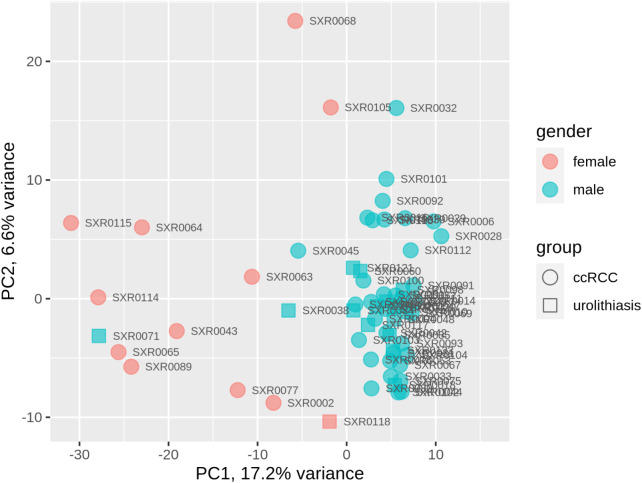
Principal component analysis plot of the expression values in the discovery cohort. Data points can be separated by gender rather than by patient group (tumor, control). PC1/PC2 – principal components 1 and 2 that explain most of the variance in the expression data

Principal component analysis (Fig. 2, Suppl. Figure S3) and a cluster analysis of the expression values of all patients (Suppl. Figure S4) were performed. Patient gender was strongly reflected by the RNA profiles, whereas tumor status (ccRCC/urolithiasis) was not. The higher principal components did not separate the samples by tumor status either (Suppl. Figure S3). Based on this observation, we decided to adjust for gender and age, a well-known risk factor for tumor onset, in the following analyses.

### Many snoRNAs were differentially expressed in ccRCC compared to urolithiasis

**Fig. 3:**
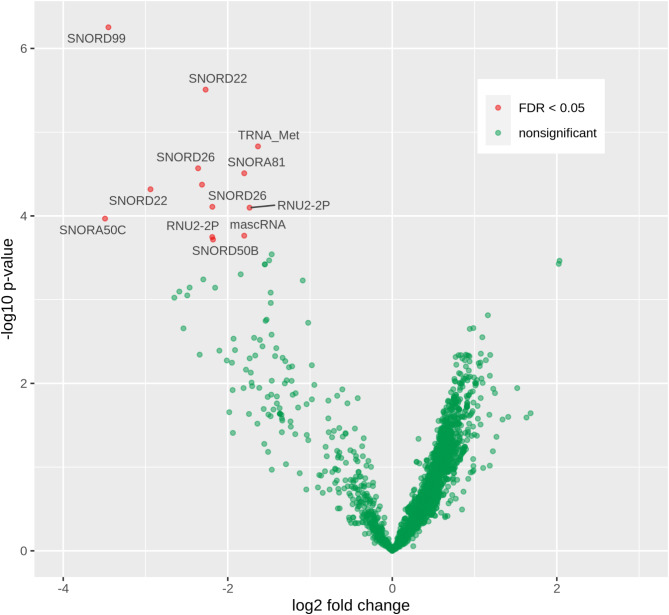
Volcano plot of the differential region expression between ccRCC and urolithiasis control patients. Each point stands for a tested region. Red dots indicate significant differences with a false discovery rate (FDR) < 0.05 adjusting for multiple testing. Horizontal axis shows strength and direction of the difference (positive values means higher expression in ccRCC compared to urolithiasis). Vertical axis shows the logarithmized p-value (higher values mean lower p-value). Genes that overlap with the significantly altered regions are annotated

**Fig. 4:**
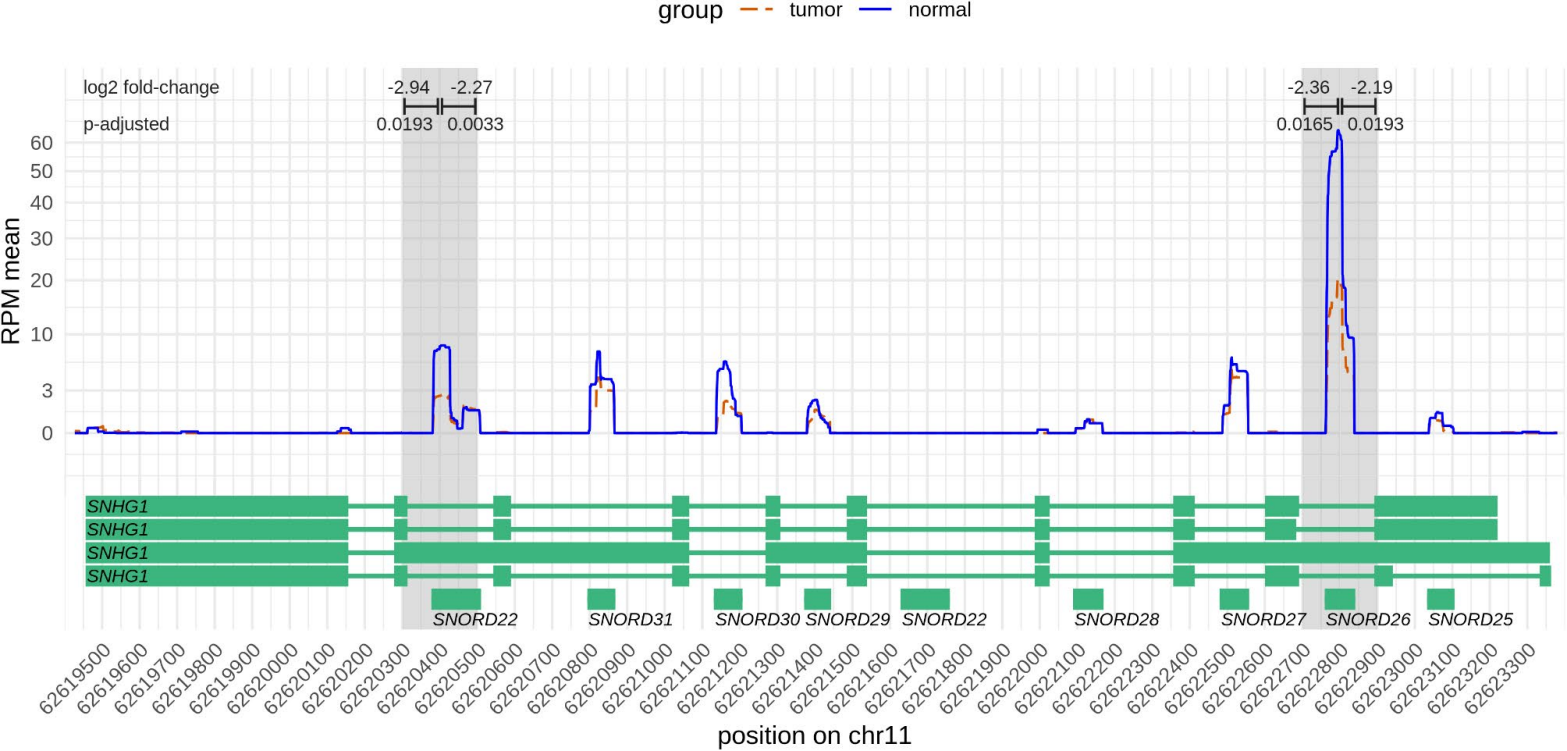
Nucleotide-wise RNA expression along *SNHG1*. The plot shows the RNA expression in the ccRCC (red dashed line) and urolithiasis (blue line) groups averaged over the patients in each group, respectively. Exon / intron structures of the transcripts of the *SNHG1* host gene are shown below. Clearly, snoRNAs within the introns are expressed instead of the exons of *SNHG1*. The significantly differentially expressed regions of *SNORD22* and *SNORD26* are indicated above the plot with log2 fold-changes and adjusted p-values

To identify urinary RNA biomarkers, we next applied DESeq2 to discover differential exosomal RNA expression at the level of genomic regions [48]. It accurately modeled read counts and employed a regression model that accounted for gender and age as confounders. Overall, 80% (4977/6234) of the regions were more highly expressed in the ccRCCs than in the controls (Fig. 3, Suppl. Table S4). Thirteen differentially expressed regions were detected, all of which were less expressed in the ccRCC-derived urinary EVs, contrary to the general trend (Table 3). Surprisingly, most of the identified regions resulted from expression of snoRNAs (*SNORD99*, *SNORD22*, *SNORD26*, *SNORA50C*, *SNORA81*, *SNORD50B*) located within introns of their respective host genes, which were barely or not at all expressed (Fig. 4, Suppl. Figures S5–S12). This was the case, for example, for *SNORD22* and *SNORD26* residing within introns of *SNHG1* (small nucleolar RNA host gene 1, Fig. 4). Looking at the genomic environment, it was observed that in some cases other snoRNAs of the host genes also appeared to have altered expression, although this was not detected to be significant. For example, this was the case for *SNORD30* in *SNHG1* and *SNORD50A* in *SNHG5* (Suppl. Figures S8 and S12). A very small fraction of the genomically large *MALAT1* was differentially expressed. Further inspection revealed the small (58 nt) *mascRNA* at the 5’ end of *MALAT1* as potentially differentially expressed in ccRCC (Suppl. Figure S9).

**Table 3:** Differentially expressed regions and their overlapping genes

Differentially expressed regions					Genes / transcripts that overlap the regions				
Chr	Start	End	Log_2_ fold change	Corrected p-value		Gene	Biotype	Description	
chr1	28,905,201	28,905,300	-3,45	0,0009		*SNORD99*	snoRNA	small nucleolar RNA, C/D box 99	
chr1	153,643,701	153,643,800	-1,63	0,0119		*TRNA_Met*	tRNA	transfer RNA Met	
chr1	153,643,801	153,643,900	-2,19	0,0256		*TRNA_Met*	tRNA	transfer RNA Met	
chr11	62,609,001	62,609,100	-2,19	0,0439		*RNU2-2P*	snRNA	RNA, U2 small nuclear 2, pseudogene	
chr11	62,609,101	62,609,200	-1,73	0,0256		*RNU2-2P*	snRNA	RNA, U2 small nuclear 2, pseudogene	
chr11	62,620,301	62,620,400	-2,94	0,0193		*SNORD22*	snoRNA	small nucleolar RNA, C/D box 22	
chr11	62,620,401	62,620,500	-2,27	0,0033		*SNORD22*	snoRNA	small nucleolar RNA, C/D box 22	
chr11	62,622,701	62,622,800	-2,36	0,0165		*SNORD26*	snoRNA	small nucleolar RNA, C/D box 26	
chr11	62,622,801	62,622,900	-2,31	0,0193		*SNORD26*	snoRNA	small nucleolar RNA, C/D box 26	
chr11	65,267,201	65,267,300	-1,80	0,0439		*MALAT1*	lincRNA	metastasis associated lung adenocarcinoma transcript 1	
chr17	62,223,801	62,223,900	-3,49	0,0314		*SNORA50C*	snoRNA	small nucleolar RNA, H/ACA box 76	
chr3	186,504,601	186,504,700	-1,80	0,0165		*SNORA81*	snoRNA	small nucleolar RNA, H/ACA box 81	
chr6	86,387,301	86,387,400	-2,18	0,0439		*SNORD50B*	snoRNA	small nucleolar RNA, C/D box 50B	

Negative log2-fold change indicates stronger expression in urolithiasis control patients. The adjusted p-values were based on the results of the regression models adjusted for gender and age of the patients, which were corrected for multiple testing (false discovery rate)

### Some genes validated by PCR were associated with increased risk of ccRCC

**Fig. 5:**
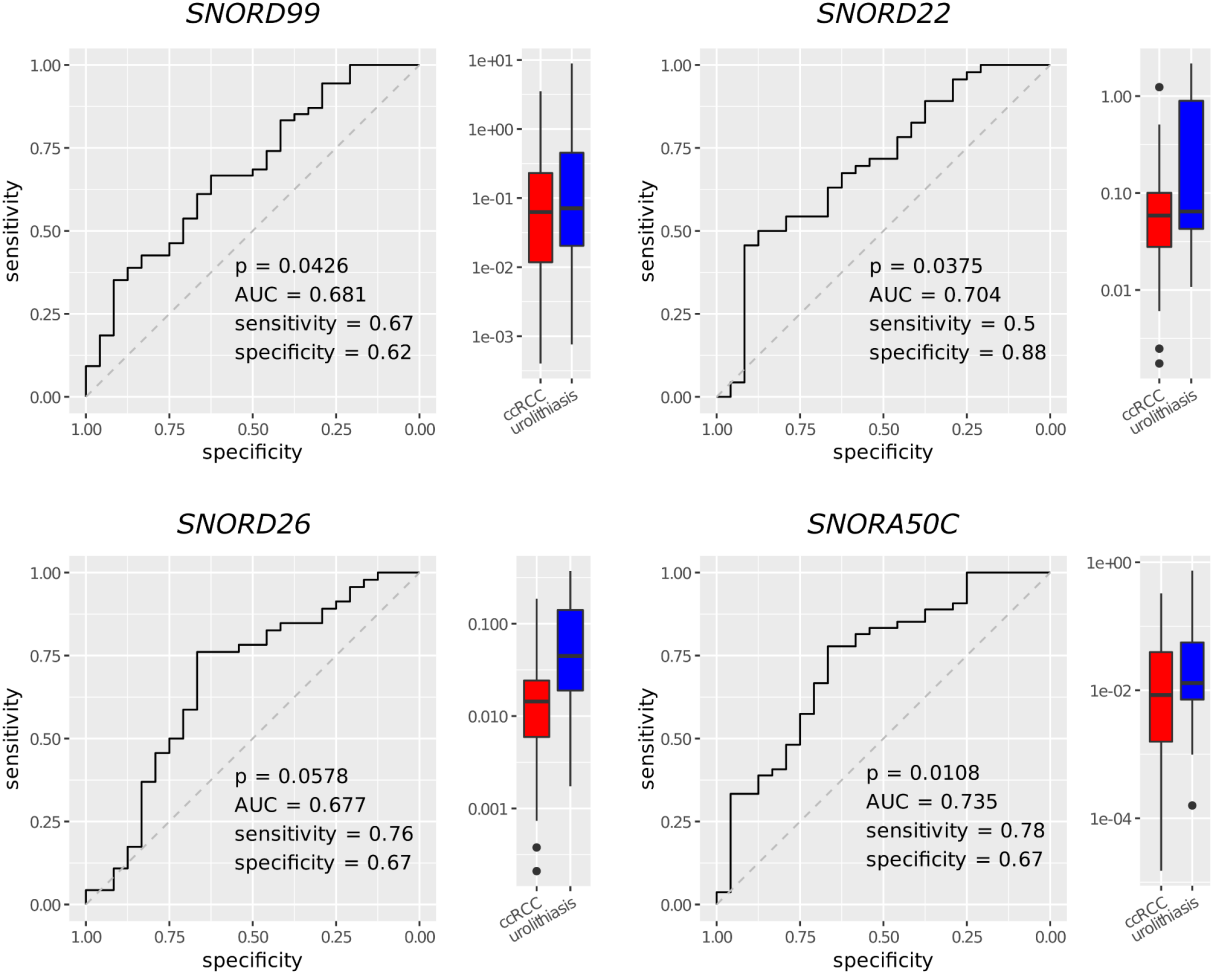
Diagnostic performance of all tested small RNAs. Receiver operating characteristic (ROC) curves with area under the curve (AUC) and boxplots of the corresponding expression values of the genes in the ccRCC and urolithiasis groups. The basis of the ROC curves were logistic regressions with RNA molecule counts normalized to the reference genes *ACTB* (*SNORD99*, *SNORA50C*) and *RNY3* (*SNORD22*, *SNORD26*). The models were adjusted for age and gender of the subjects

Next, genes were selected for validation by qPCR (Fig. 5; Table 4) in the larger validation cohort (54 ccRCC and 24 control subjects, including all discovery cohort subjects). Some genes were too short for qPCR assay design (*mascRNA*, *SNORD50B*), or failed (*SNORA81*). *RNY3* and *ACTB* showed consistent expression in the RNA-Seq data and were selected as reference genes for qPCR. Expression of all candidate genes was lower in ccRCC compared to urolithiasis, confirming the observations in the RNA-seq data. Regression models adjusted for age and gender were used to assess the predictive power for discriminating ccRCC patients from urolithiasis controls (Table 4; Fig. 5). The area under the curves (AUCs) of all genes were moderately high (0.677–0.735) and accuracy ranged from 0.629 to 0.744 (upper half of Table 4). Odds ratios were calculated and showed that lower expression in EVs was significantly associated with the occurrence of ccRCC in almost all cases and marginally significant for *SNORD26* (*p* = 0.0578). Moreover, we combined the four snoRNAs in pairs, groups of three and four genes in order to investigate possible complementary and enhancing effects. This lead to improvements of the diagnostic performance. Particularly the combinations *SNORD22* + *SNORA50C*, *SNORD99* + *SNORD22* + *SNORA50C* and *SNORD99* + *SNORD22* + *SNORD26* + *SNORA50C* had better accuracies and AUCs than the models with the corresponding genes alone (Table S5). Six of 11 models were significantly better than the null-model (*p* < 0.05, chi-squared test), the other five models were marginally significant (*p* < 0.08).

**Table 4:** Areas under the curve (AUC), sensitivities, specificities, accuracies and p-values of the regression models

Combination of genes and clinical risk factors	Sensitivity	Specificity	Accuracy	AUC	OR_IQR_	p
*SNORD99*	0.667	0.625	0.654	0.681	0.384	**0.0426**
*SNORD22*	0.500	0.875	0.629	0.704	0.560	**0.0375**
*SNORD26*	0.761	0.667	0.729	0.677	0.573	0.0578
*SNORA50C*	0.778	0.667	0.744	0.735	0.210	**0.0108**
*SNORD99* + OBS + HTN	0,900	0,417	0,743	0,693	0.385	0.0657
*SNORD22* + OBS + HTN	0,791	0,667	0,746	0,733	0.570	0.0586
*SNORD26* + OBS + HTN	0,628	0,750	0,672	0,720	0.541	**0.0495**
*SNORA50C* + OBS + HTN	0,840	0,625	0,770	0,766	0.165	**0.0046**

The expression values of the genes validated with qPCR were used in the regression models alone or in combination with obesity (OBS; BMI ≥ 30) and hypertension (HTN) as clinical risk factors. All models were adjusted for age and gender. The p-values of the top four results are those of the genes in the models (association with the response). The p-values of the bottom four results are those of the overall model fit (chi-squared test against the null model). P-values < 0.05 are shown in bold. Odds ratios (OR) for the risk of ccRCC presence were calculated on the basis of a gene expression change in the size of the interquartile range (IQR) of the respective gene

Fivefold cross-validation was performed to assess the robustness of the trained models (Suppl. Table S6). It showed that the generalized performance of most models was slightly reduced, but in a similar range to that of the models trained on the full dataset. For example, the accuracy range of the single-gene models was 0.629–0.735 on the full dataset, and 0.640–0.725 during cross-validation. The range of accuracy decreased from 0.671 to 0.757 to 0.624–0.705 for the two-gene models.

We also tested whether the inclusion of further ccRCC risk factors could enhance the diagnostic performance of the single-gene models. Accordingly, information on hypertension and obesity was available for the patients of our cohort. Inclusion of both risk factors improved almost all accuracies and AUCs of the models (lower half of Table 4). In addition, including these two risk factors also improved all accuracies and AUCs of the models with two, three and four gene combinations. Ten of the fifteen models with the additional risk factors were significant (*p* < 0.05, chi-squared test against the null model). The highest accuracy (0.811) and AUC value (0.773) was obtained by the inclusion of *SNORD99*, *SNORA50C*, obesity and hypertension (*p* = 0.0091, Table S5). *SNORA50C* provided the best overall performance in terms of accuracy, p-value and robustness, for both the single-gene and multi-gene models.

## Discussion

CcRCC is a frequent tumor with a low survival rate [4]. Newly detected cases often metastasize [3] due to their asymptomatic behavior in the early stage [2]. Hence, there is an urgent need for biomarkers for the early detection of ccRCC. In this study, we have screened for urine-derived biomarkers in a cohort of 47 ccRCC patients and 16 controls with urolithiasis. Since previous biomarker studies reported findings of small RNA, we used a small RNA-seq protocol. Cluster analysis showed a clear separation into male and female, while the tumor status was not reflected globally. Therefore, we decided to adjust for gender and age in the expression screening and found 13 differentially expressed regions in nine genes, mainly from snoRNA species. Four of them (*SNORD99*, *SNORD22*, *SNORD26*, *SNORA50C*) were validated by qPCR as potential biomarkers for ccRCC in an extended cohort of 54 ccRCC and 24 urolithiasis patients. All candidates showed moderate sensitivity, specificity and a higher risk of ccRCC at lower expression. The combination of the snoRNAs and the inclusion of further RCC risk factors [19] into the regression models clearly increased the diagnostic performance. The model with the snoRNAs *SNORA50C*, *SNORD99* and hypertension and obesity as additional RCC risk factors showed the best diagnostic performance (accuracy = 0.811, AUC = 0.733, *p* = 0.0091). Therefore, these snoRNAs might be potential, new biomarkers for the early, urine-based detection of ccRCC in a convenient, non-invasive way.

In addition to other snoRNAs, the candidate *SNORD99* is located in an intron of *SNHG12*, a gene which is upregulated in RCC and associated with poor prognosis [51, 52]. As a competing endogenous RNA (ceRNA), it can sponge different miRNAs thereby regulating their target genes [53, 54]. Higher *SNHG12* expression in RCC cell lines correlated with proliferation, migration and invasion of tumor cells [51, 53]. In our study, *SNORD99* showed reduced levels in urinary EVs of the ccRCC patients. Interestingly, it was reported to be decreased in colon cancer but increased in immune cells associated with tumor infiltration [55].

Both *SNORD22* and *SNORD26* are located in introns of *SNHG1*, whose suppression can also reduce that of its intronic snoRNAs [56]. *SNORD22* showed higher levels in serum exosomes of pancreatic cancer patients compared to healthy controls [57]. *SNORD26* is among the top differentially expressed snoRNAs in prostate cancer [58] and is strongly associated with immune response and survival in low-grade glioma [59]. *SNHG1* itself is also known to sponge miRNAs in various cancers, including RCC, where it promotes proliferation, invasion, metastasis formation and immune escape [60–62].

*SNORA50C* (alias *SNORA76*) was significantly upregulated in gallbladder cancer vs. matched adjacent non-tumor tissues [63] and significantly downregulated in metastatic vs. non-metastatic prostate cancer xenograft models [64]. *SNORA50C* contributed to cell growth and migration through the *HDAC1*-mediated pathway in neuroblastoma, where its depletion suppressed tumor cell proliferation, invasion and migration [65]. Taken together, the literature suggests that all of the candidate genes found are involved in diverse cancers, including RCC. This supports their suitability as screening targets and provides insight into their mode of action.

Several studies have explored liquid biopsies for the diagnosis and prognosis of ccRCC before. Mainly, EVs from the blood of ccRCC patients have been analyzed for the abundance of known miRNAs [33, 66] or screened to discover new miRNA biomarkers [67, 68]. Recently, other RNA species have been found to have diagnostic potential too. For example, Zhao et al. identified a signature of six snoRNAs in serum that distinguished ccRCC patients from healthy controls with an AUC value of 0.75 [69]. Urine also holds great promise for biomarker discovery because it is produced in the kidney and easy to collect. Two studies found that cell-free miRNAs from patient urine had diagnostic potential for RCC and ccRCC in particular [70, 71], reporting AUCs of 0.83 (*let-7a*) and 0.96 (*miRNA-15a*), respectively. In addition, snoRNAs have also been discovered in urine samples from RCC patients. For example, *SNORD63* and *SNORD96A* were described as potential biomarkers from urine sediments of ccRCC patients with AUC values of 0.71 and 0.68, respectively [72].

SnoRNAs are typically 60–300 nt in length and are associated with ribonucleoproteins. Their major cellular functions include the pre-rRNA maturation, 2’-O-methylation and pseudouridylation of target molecules, as well as binding competition, protein trapping and factor recruitment [73, 74]. Their expression is controlled by their host genes, copy number variations and DNA methylation, which is frequently altered in many tumor entities, including RCC, ultimately leading to deregulation of a variety of cellular processes [38, 73]. Processes such as metabolic reprogramming, alteration of the tumor microenvironment, and enhancement of tumor cell proliferation, migration and invasion are critically involved in the onset and progression of ccRCC [75, 76]. In part, these may be due to changes in snoRNA expression and function. The importance of this class of regulatory RNAs in RCC is further supported by the identification of a snoRNA-specific transcript cluster in advanced RCC [77]. Thus, it is reasonable to expect that snoRNAs can reflect RCC biology and may therefore serve as biomarkers for this tumor entity, as shown here and in previous studies [69, 72].

Although cell-free miRNAs and snoRNAs have been detected in liquid biopsies from patients, RNA from EVs is likely to provide more reliable biomarkers because the cargo of microvesicles is better protected from degradation. Early work on urine-derived EVs yielded transcript biomarkers such as mRNAs (*GSTA1*, *CEBPA*, *PCBD1*) and miRNAs (miR-126-3p, miR-449a) for ccRCC detection [37, 78]. The latter reported an AUC of 0.84 and 0.79 to discriminate ccRCC from healthy subjects, respectively. Moreover, miR-30c-5p was identified by RNA-seq as a urinary EV-derived biomarker that discriminated ccRCC from healthy subjects (AUC 0.82) [35]. Another RNA-seq study found many non-coding RNAs, including tRNA, miRNAs and lincRNAs, as potential urine-derived EV biomarkers for chronic kidney disease [79]. To the best of our knowledge, urinary EV-derived snoRNAs identified by NGS were reported here for the first time for ccRCC diagnosis.

The snoRNAs discovered in our study as potential ccRCC biomarkers had AUC values between 0.68 and 0.74 in the single-gene models. Although these are only moderate values, they could help to improve RCC diagnosis. Unlike in most other studies, the models used here were adjusted for patient age and gender. Both parameters are known risk factors for RCC onset and should therefore be considered as model cofactors [19]. Gender can be reflected in global expression patterns as observed in our and in other studies [80]. Adjusting for both clinical parameters allows to more accurately determine the true diagnostic value of the proposed expression markers. Moreover, the use of information on obesity and hypertension, two other known risk factors for RCC [19], improved the diagnostic performance. Both types of information are usually readily available from individuals consulting a urologist and are therefore practical for screening purposes.

To improve diagnostic performance, we also tested combinations of two, three or all four genes as model predictors, similar to what was done in other studies [37, 69]. In most cases, a slight improvement in performance over the single-gene models was observed. A further, clear predictive benefit was observed when the multi-gene models were combined with the additional risk factors of hypertension and obesity. Future studies may investigate other types of cargo in extracted EVs, such as tumor-associated proteins, for their potential to further improve diagnosis. This will require the development of integrative protocols that are easily applicable. First steps in this direction have already been taken [81].

Only three studies with similar settings were found for potential validation (GSE125442: Liu et al., 2019, unpublished) [35, 78]. However, our candidate snoRNAs were almost completely absent from these data, so validation could not be performed. As a substitute, we estimated the generalization performance of the models through fivefold cross-validation. This showed that the generalization performance was slightly reduced, but still very similar to the performance of the models trained on the full datasets.

A special feature of our study is that urolithiasis patients were used as controls instead of healthy subjects. With this choice we wanted to simulate the real clinical situation in which patients with similar symptoms undergo urological diagnosis. A comparison with healthy subjects would not reflect this situation. However, this different type of control group results in a lack of comparability with previous studies, corroborated by a recent study that indicated a divergent miRNA pattern in urinary EV between healthy subjects and those with urolithiasis [82]. Furthermore, most RNA-seq-based studies of urinary exosomes have focused on mRNA or miRNA signatures rather than the comprehensive consideration of all small RNA species. Thus, most studies provided completely different biomarker sets. This shows that the reliability and accuracy of diagnostic tools depend on the optimization of laboratory and computational protocols. Consistently, recent reviews conclude that still much time and effort is needed before EV-based biomarkers can be widely used for diagnosis [83, 84].

## Conclusions

We reported here four snoRNAs as promising biomarkers from urine-derived EVs for the non-invasive detection of ccRCC. They allow to discriminate ccRCC from urolithiasis patients with moderate accuracy independent of subject age and gender. These biomarker candidates could contribute to the development of new, easily applicable diagnostic tools for the early detection and monitoring of ccRCC.

### Electronic supplementary material

Below is the link to the electronic supplementary material.

Supplementary Material 1

Supplementary Table S1

Supplementary Table S4

## Data Availability

All data generated and analyzed during this study are included in this published article, its supplementary information files or online at Zenodo (10.5281/zenodo.10654767). RNA-seq data are not publicly available to protect the privacy of study participants. All original code has been deposited at github (https://github.com/konradgrutz/ccRCC_biomarker_analysis, 10.5281/zenodo.10654813).

## Abbreviations

AUC: area under the curve
BMI: body mass index
ccRCC: clear cell renal cell carcinoma
CI: confidence interval
CT: computed tomography
CV: coverage variance
EV: extracellular vesicle
FDR: false discovery rate
HTN: hypertension
IQR: interquartile range
lincRNA: long intergenic non-coding RNA
miRNA: micro-RNA
nt: nucleotide
OBS: obesity
OR: odds ratio
PC: principal component
PCA: principal component analysis
qPCR: quantitative polymerase chain reaction
RCC: renal cell carcinoma
rlogTPM: regularized log transformation of transcripts per kilobase million
RNA-seq: RNA sequencing
ROC: receiver operating characteristics
SD: standard deviation
snoRNA: small nucleolar RNA
snRNA: small nuclear RNA
TPM: transcripts per kilobase million
