# Supplementary material for: Identification of novel snoRNA-based biomarkers for clear cell renal cell carcinoma from urine-derived extracellular vesicles
Source: Biol Direct. 2024 May 13;19:38. doi: 10.1186/s13062-024-00467-0 (PMC11089706; doi:10.1186/s13062-024-00467-0)
Supplement: Supplementary file 1 — Supplementary Material 1 [file 13062_2024_467_MOESM1_ESM.pdf]

Supplementary Figures

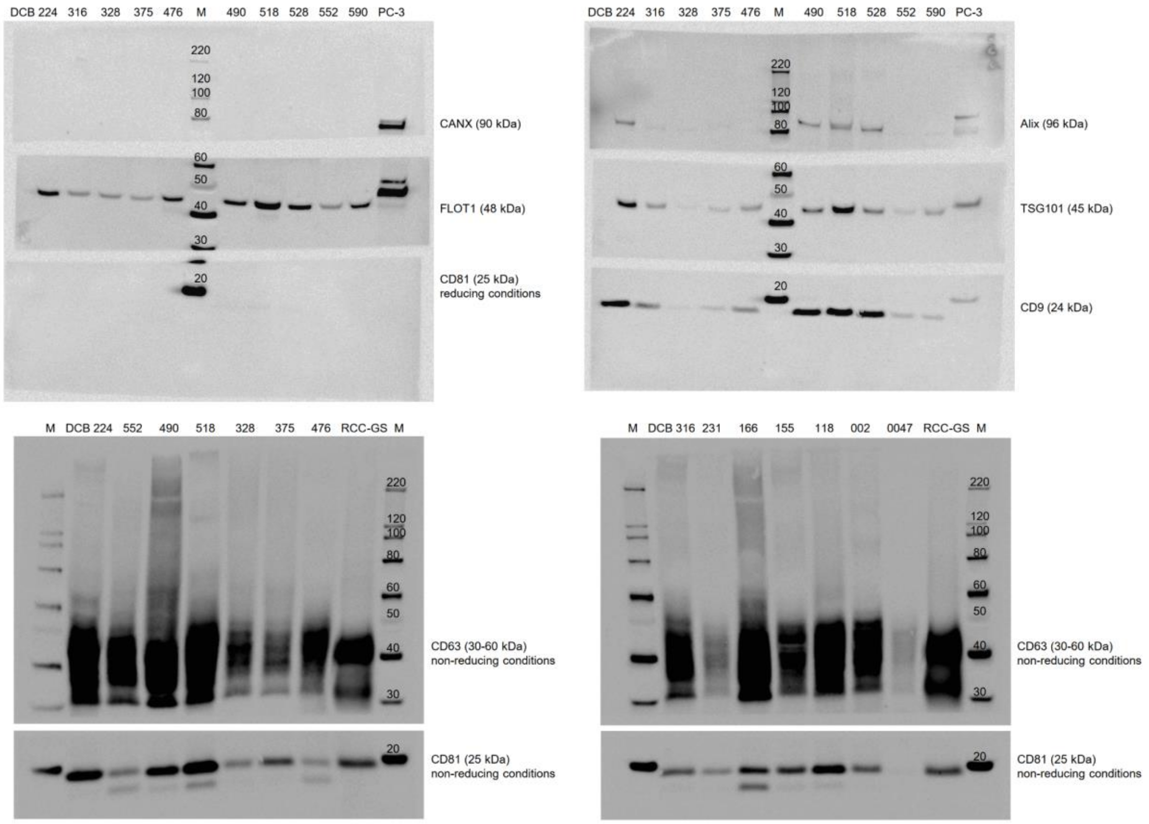

Suppl. Figure S1: Western blots of proteins extracted from the enriched EVs. The blots for different exemplary EV extraction samples (DCB + number) indicate that mainly exosomes were enriched according to the detection of the exosomal markers Alix, CD9, CD63, CD81, FLOT1 and TSG101. Calnexin (CANX) as marker of the endoplasmic reticulum served as negative control, which should be absent in exosome preparations. The cell lines PC3 and RCC-GS were used as positive controls for the different proteins. M was the size marker.

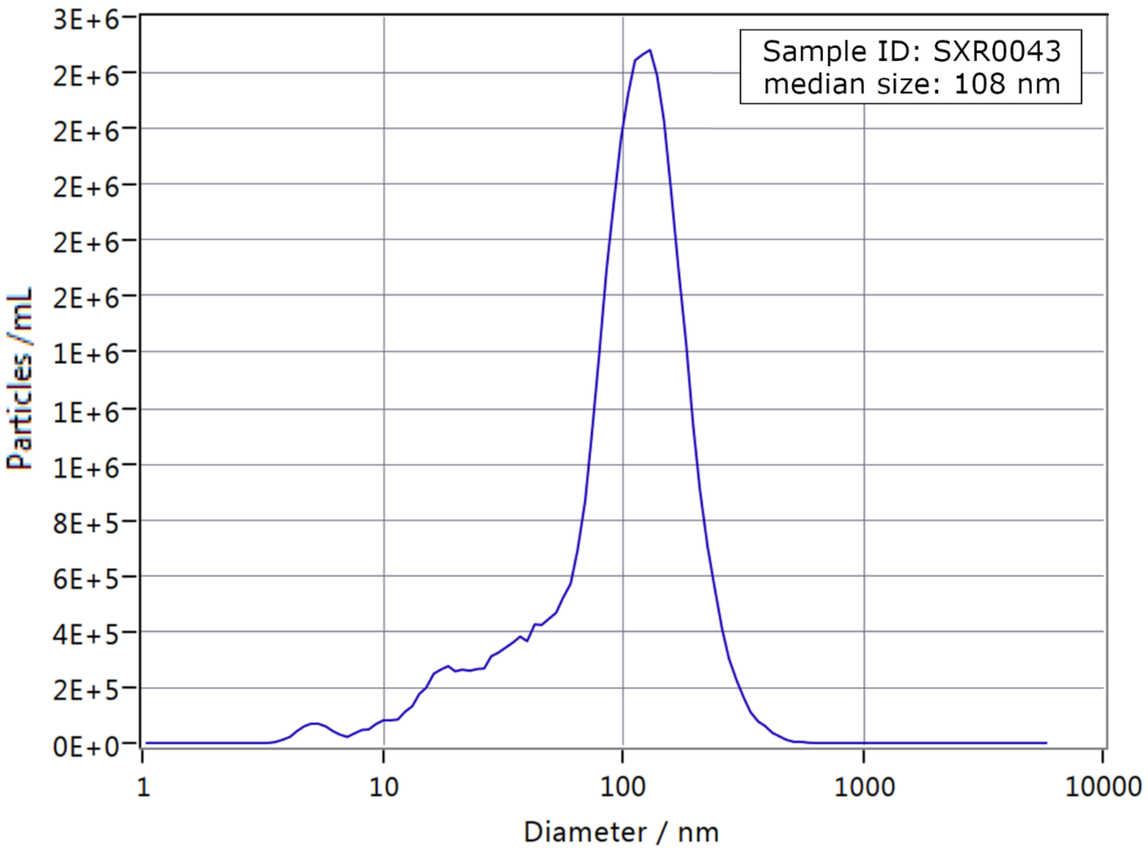

Suppl. Figure S2: Characterization of urinary EVs by nano-tracking analyses. Measurements with the Zeta View device assessed the distribution of the size and concentration of the enriched microvesicles as shown exemplarily for urine sample SXR0043. The typical peak around 110-120 nm indicated that mainly exosomes have been enriched.

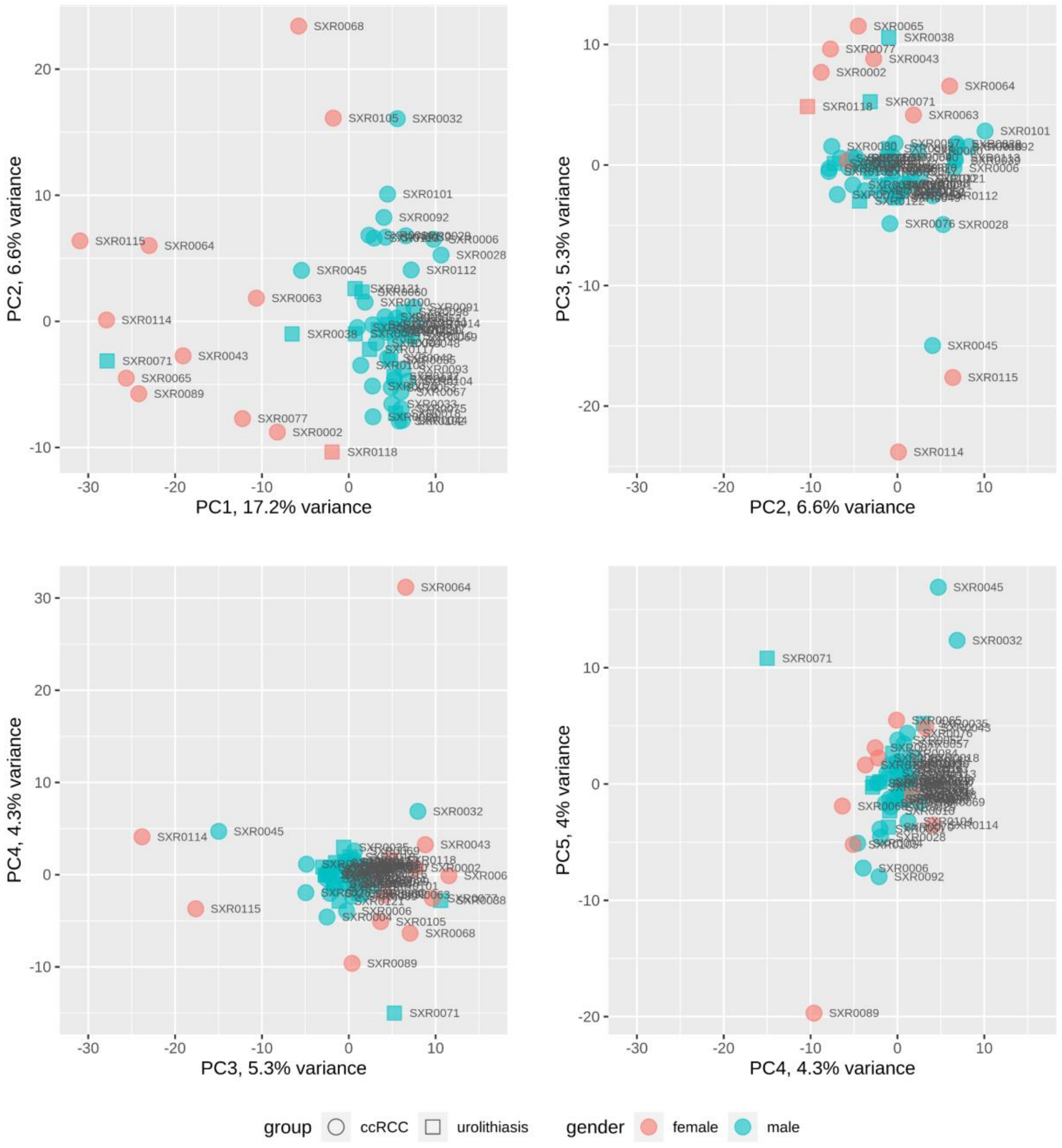

Suppl. Figure S3: **Principal component analyses plots of the expression values in the discovery cohort.** The basis were regularized logarithmic transformed expression values. PC1-PC5 - principal components 1 to 5 and their percentages of explained expression variance.

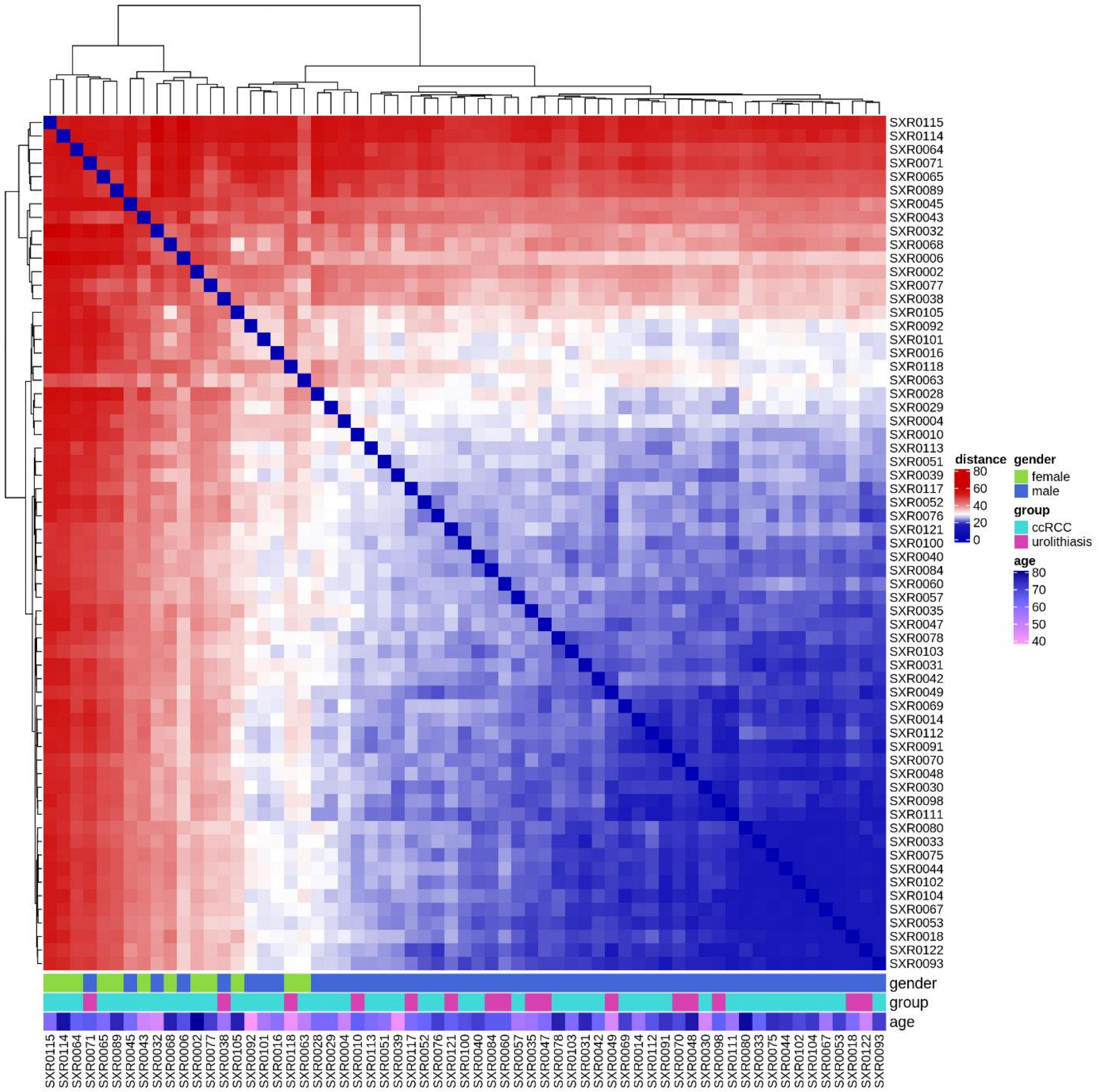

Suppl. Figure S4: Heatmap from hierarchical clustering of genomic region expression of all samples. Clustering was based on Euclidian sample distances of the regularized logarithmic transformed expression values. Each row and column denotes a sample. The dendrogram shows that the samples are split into two groups with mainly male and female patients, respectively, while there is no separation according to ccRCC and urolithiasis.

Suppl. Figures S5-S12: Nucleotide-wise RNA expression around the differentially expressed region. Plot shows the RNA expression in the ccRCC (red) and urolithiasis (blue) groups averaged over the patients of each group, respectively. Exon / intron structures of the overlapping genes are shown below the expression graphs. Clearly, in most cases, snoRNAs within introns are expressed and secreted via EVs instead of the exons of their host genes. The significantly differentially expressed regions are highlighted with light blue vertical bars. The plots are customized screenshots from UCSC genome browser.

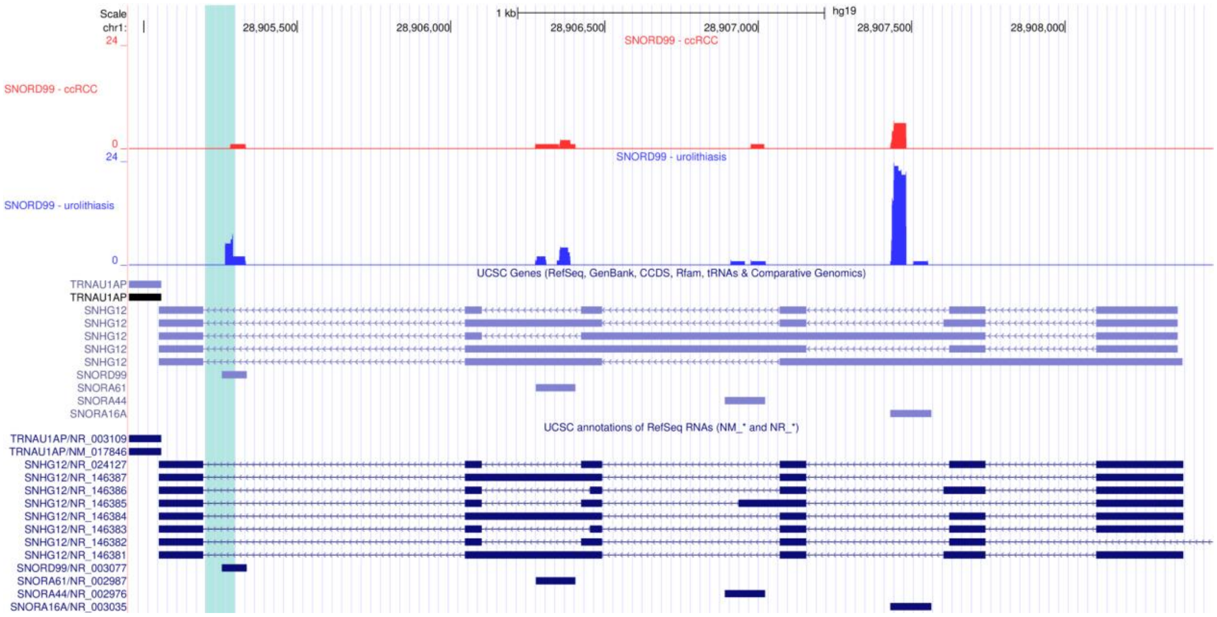

Suppl. Figures S5: ***SNHG12 / SNORD99***

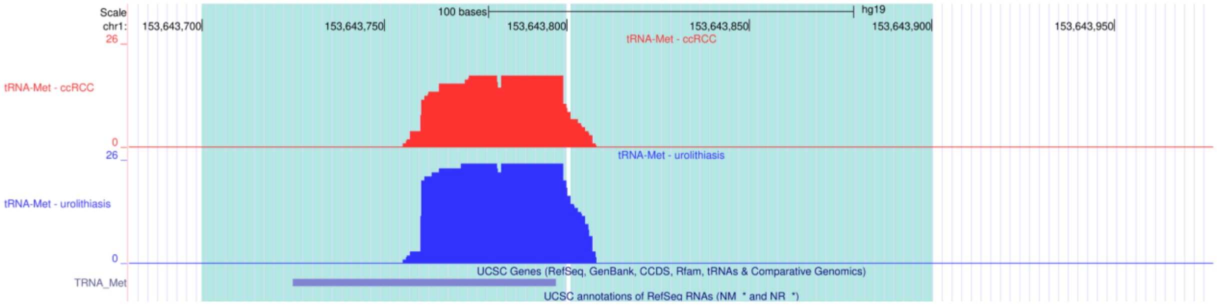

Suppl. Figure S6 – tRNA-Met

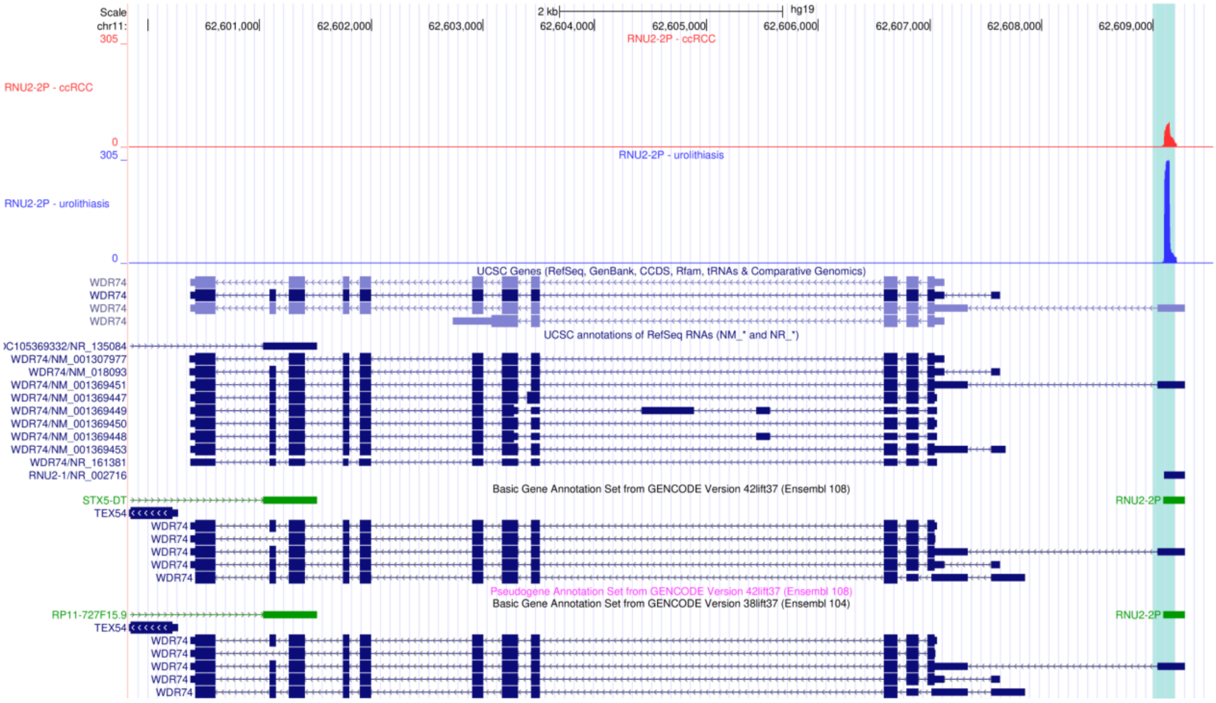

Suppl. Figure S7 – *WDR74* / *RNU2-2P*

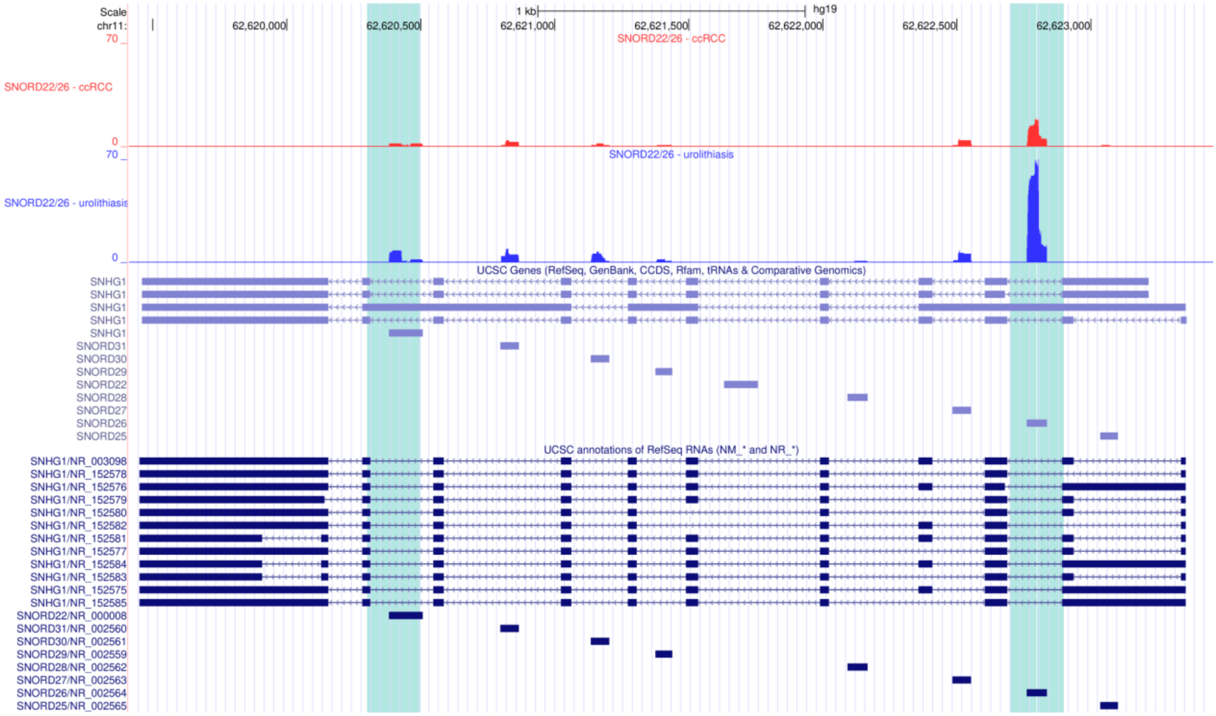

Suppl. Figure S8 – *SNHG1* / *SNORD22* / *SNORD26*

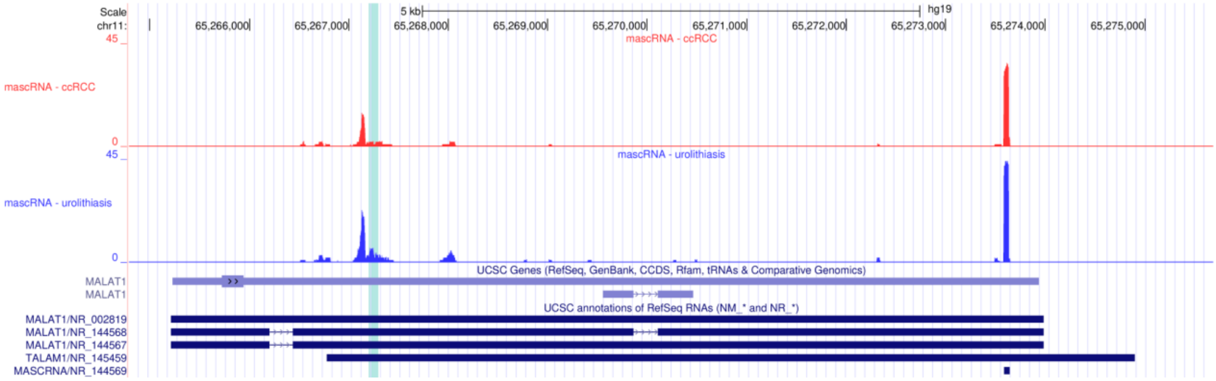

Suppl. Figure S9 - *MALAT1 / mascRNA*

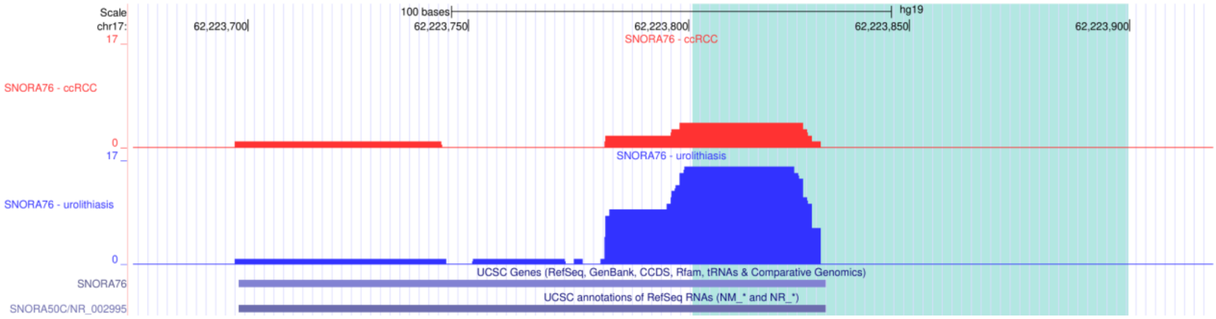

Suppl. Figure S10 – *SNORA50C* (alias *SNORA76*)

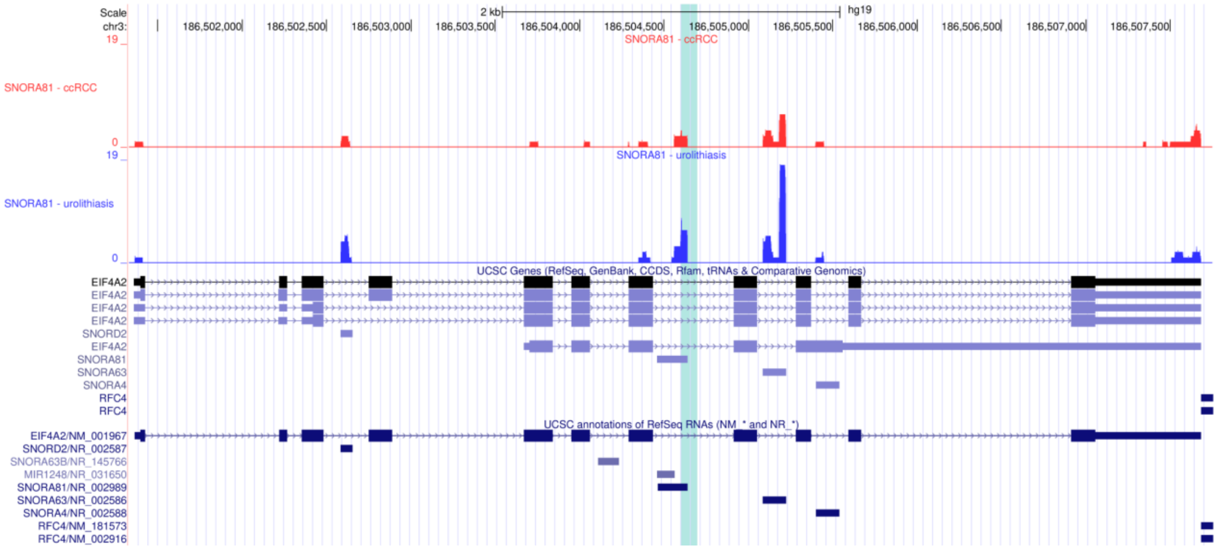

Suppl. Figure S11 - *EIF4A2 / SNORA81*

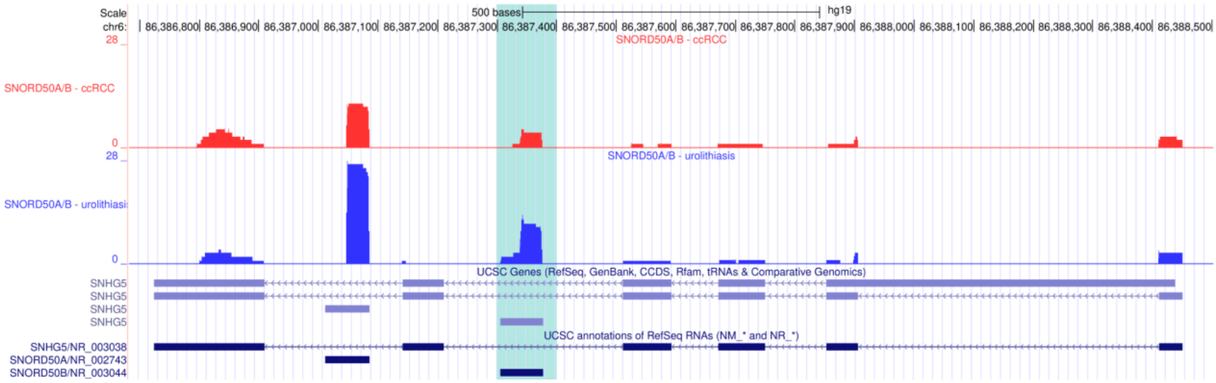

Suppl. Figure S12 - *SNHG5 / SNORD50B*

Supplementary Tables

Suppl. Table S1: Information on the entire patient cohort.

[external file]

Given are RNA quality parameters, information on DNA disturbance, demographic variables, pathological characteristics and parameters for the urine sampling.

Suppl. Table S2: Antibodies used for assessment of exosomal markers by Western blot.

Target	Antibody	Clone	Provider	Dilution
Alix	Purified anti-Alix Antibody	3A9	BioLegend	1:500
CANX	Calnexin Rabbit mAb	C5C9	Cell Signaling	1:1000
CD9	Anti-CD9 antibody	EPR2949	Abcam	1:500
CD63	Exosome – anti-CD63	TS63	Thermo Fisher	1:5000
CD81	Exosome – anti-CD81	M38	Thermo Fisher	1:250
FLOT1	Purified Mouse Anti-Flotillin-1	18/Flotillin-1	BD Biosciences	1:250
TSG101	TSG101 antibody	4A10	GeneTex	1:1000
anti-mouse	Rabbit Anti-Mouse Immuno-globulins / HRP	P0260 (polyclonal)	DAKO	1:1000
anti-rabbit	Swine Anti-Rabbit Immuno-globulins / HRP	P0217 (polyclonal)	DAKO	1:1000

Suppl. Table S3: Taqman Assays for validation by qPCR (all from Thermo Fisher Scientific).

Gene	Gene type	Chro-mosome	Start	End	Strand	Assay ID	Assay type
SNORA50C	target gene	chr17	62,223,699	62,223,831	+	Hs03298703_s1	non-coding RNA assay
SNORA81	target gene	chr3	186,504,465	186,504,641	-	CTNKRVK	custom gene expression assay
SNORD22	target gene	chr11	62,620,382	62,620,507	-	CTKA3PR	custom gene expression assay
SNORD26	target gene	chr11	62,622,764	62,622,838	-	CTMFXAN	custom gene expression assay
SNORD99	target gene	chr1	28,905,255	28,905,334	-	Hs03309518_s1	gene expression assay
ACTB	reference gene	chr7	5,566,779	5,570,232	-	Hs99999903_m1	gene expression assay
RNY3	reference gene	chr7	148,680,847	148,680,948	+	CTWCWRE	custom gene expression assay

Genome coordinates are based on hg19.

Suppl. Table S4: Results of the tests for differential region expression comparing ccRCC against urolithiasis patients.

[external file]

Tests were adjusted for patient age and gender. Given are the chromosomal position of the tested regions, the overlapping genes and their annotation, the results of the differential expression analysis, where FDR is the false discovery rate derived from the p-values by Benjamini-Hochberg correction, the expression counts for each sample and the mean and median expression counts per patient group, each.

Suppl. Table S5: Results of the regression models testing combinations of two, three or four genes and clinical risk factors.

Combination of genes and clinical risk factors	Sensitivity	Specificity	Accuracy	AUC	P-value
SNORD99 + *SNORD22*	0.587	0.875	0.686	0.714	0.0595
SNORD99 + *SNORD26*	0.848	0.542	0.743	0.694	0.0713
SNORD99 + *SNORA50C*	0.778	0.667	0.744	0.735	**0.0101**
SNORD22 + *SNORD26*	0.609	0.792	0.671	0.699	0.0591
SNORD22 + *SNORA50C*	0.804	0.667	0.757	0.734	**0.0168**
SNORD26 + *SNORA50C*	0.783	0.583	0.714	0.729	**0.0227**
SNORD99 + *SNORD22* + OBS + HTN	0.837	0.625	0.761	0.734	0.0629
SNORD99 + *SNORD26* + OBS + HTN	0.977	0.417	0.776	0.729	0.0572
SNORD99 + *SNORA50C* + OBS + HTN	0.900	0.625	0.811	0.773	**0.0091**
SNORD22 + *SNORD26* + OBS + HTN	0.744	0.667	0.716	0.727	**0.0480**
SNORD22 + *SNORA50C* + OBS + HTN	0.907	0.583	0.791	0.762	**0.0066**
SNORD26 + *SNORA50C* + OBS + HTN	0.930	0.583	0.806	0.766	**0.0073**
SNORD99 + *SNORD22* + *SNORD26*	0.630	0.750	0.671	0.710	0.0789
SNORD99 + *SNORD22* + *SNORA50C*	0.826	0.667	0.771	0.738	**0.0331**
SNORD99 + *SNORD26* + *SNORA50C*	0.783	0.583	0.714	0.728	**0.0445**
SNORD22+ *SNORD26* + *SNORA50C*	0.804	0.625	0.624	0.730	**0.0314**
SNORD99 + *SNORD22* + *SNORD26* + OBS + HTN	0.977	0.417	0.776	0.730	0.0656
SNORD99 + *SNORD22* + *SNORA50C* + OBS + HTN	0.907	0.583	0.791	0.770	**0.0109**
SNORD99 + *SNORD26* + *SNORA50C* + OBS + HTN	0.930	0.542	0.791	0.772	**0.0126**
SNORD22+ *SNORD26* + *SNORA50C* + OBS + HTN	0.930	0.542	0.791	0.765	**0.0113**
SNORD99 + *SNORD22*+ *SNORD26* + *SNORA50C*	0.826	0.625	0.757	0.736	0.0553
SNORD99 + *SNORD22*+ *SNORD26* + *SNORA50C* + OBS + HTN	0.907	0.542	0.776	0.770	**0.0172**

Sensitivities, specificities, accuracies, areas under the curve (AUC) and p-values of the logistic regression models. The model formulas contained the given combinations of genes validated with qPCR and optionally the risk factors obesity (OBS: BMI ≥ 30) and hypertension (HTN). All models were also adjusted for gender and age. P-values are those of the overall model fit (chi-squared test against the null model). P-values < 0.05 are shown in bold.

Suppl. Table S6: Results of the fivefold cross-validation.

Combination of genes	Sensitivity		Specificity		Accuracy		Area under the curve		Model p-value	
	mean	SD	mean	SD	mean	SD	mean	SD	mean	SD
SNORD99	0.650	0.267	0.618	0.271	0.640	0.199	0.649	0.120	0.082	0.082
SNORD22	0.666	0.257	0.669	0.267	0.666	0.185	0.662	0.126	0.120	0.111
SNORD26	0.655	0.284	0.621	0.250	0.642	0.211	0.650	0.122	0.146	0.124
SNORA50C	0.737	0.228	0.699	0.231	0.725	0.166	0.701	0.128	**0.019**	0.025
SNORD99 + *SNORD22*	0.642	0.275	0.627	0.276	0.637	0.203	0.646	0.123	0.126	0.115
SNORD99 + *SNORD26*	0.650	0.310	0.574	0.237	0.624	0.228	0.642	0.122	0.139	0.121
SNORD99 + *SNORA50C*	0.712	0.233	0.690	0.237	0.705	0.175	0.691	0.128	**0.037**	0.045
SNORD22 + *SNORD26*	0.655	0.284	0.636	0.256	0.648	0.207	0.656	0.126	0.123	0.114
SNORD22 + *SNORA50C*	0.707	0.262	0.661	0.244	0.690	0.189	0.677	0.128	0.052	0.061
SNORD26 + *SNORA50C*	0.706	0.265	0.642	0.245	0.683	0.191	0.671	0.129	0.063	0.068
SNORD99 + *SNORD22* + *SNORD26*	0.644	0.304	0.584	0.254	0.624	0.221	0.639	0.120	0.150	0.132
SNORD99 + *SNORD22* + *SNORA50C*	0.686	0.275	0.647	0.247	0.671	0.198	0.667	0.126	0.081	0.087
SNORD99 + *SNORD26* + *SNORA50C*	0.692	0.272	0.628	0.244	0.669	0.198	0.660	0.125	0.097	0.096
SNORD22 + *SNORD26* + *SNORA50C*	0.684	0.287	0.629	0.248	0.665	0.207	0.664	0.126	0.078	0.082
SNORD99 + *SNORD22* + *SNORD26* + *SNORA50C*	0.675	0.293	0.612	0.251	0.653	0.209	0.653	0.124	0.110	0.111

Given are the means and standard deviations (SD) of the most important model performance metrics. Cross-validation was performed for each model by randomly dividing the validation cohort into five bins, each with at least 10 ccRCC and four urolithiasis patients. The model was then trained on four data bins and tested on the one remaining data bin. Test and training were repeated over all possible combinations of the divided data. The whole procedure was repeated 1000 times for each model. P-values are those of the overall model fit (chi-squared test against the null model). P-values < 0.05 are shown in bold.
